# Optimal timing for antimicrobial prophylaxis to reduce surgical site infections: a retrospective analysis of 531 patients

**DOI:** 10.1038/s41598-023-36588-1

**Published:** 2023-06-09

**Authors:** Christoph Paasch, Claus Schildberg, Sebastian Lünse, Sophie Heisler, Jens Meyer, Jette Kirbach, Elisa Kobelt, Richard Hunger, Isabel-Elena Haller, Chrissanthi Helmke, Rene Mantke

**Affiliations:** 1 Department of Surgery, Brandenburg Medical School, University Hospital Brandenburg/Havel, 14770 Brandenburg, Germany; 2 Clinic for General and Visceral Surgery, Klinikum Magdeburg gGmbH, Magdeburg, Germany; 3 Faculty of Health Science Brandenburg, Brandenburg Medical School, University Hospital Brandenburg/Havel, 14770 Brandenburg, Germany; 4 Clinic for General and Visceral Surgery, University Hospital Brandenburg an der Havel, Brandenburg Medical University, Hochstraße 29, 14770 Brandenburg an der Havel, Germany

**Keywords:** Gastroenterology, Medical research

## Abstract

It has been revealed that the administration of an antimicrobial prophylaxis (AP) reduces the rate of surgical site (SSI) following colorectal cancer surgery. Nevertheless, the optimal timing of this medication remains unclear. The aim of this study was to determine more precisely the optimal time for administering antibiotics and to see if this could reduce the number of possible surgical site infections. The files of individuals who underwent colorectal cancer surgery at the University Hospital Brandenburg an der Havel (Germany) between 2009 and 2017 were analyzed. Piperacillin/tazobactam, cefuroxime/metronidazole and mezlocillin/sulbactam were administered as AP regimens. Timing of AP was obtained. The primary objective was the rate of SSIs based on CDC criteria. Multivariate analysis took place to identify risk factors for SSIs. A total of 326 patients (61.4%) received an AP within 30 min, 166 (31.3%) between 30 and 60 min, 22 (4.1%) more than 1 h before surgery, and 15 (2.8%) after surgery. In 19 cases (3.6%) a SSI occurred during hospital stay. A multivariate analysis did not identify AP timing as a risk factor for the occurrence of SSIs. With significance, more surgical site occurrences (SSO) were diagnosed when cefuroxime/metronidazole was given. Our results suggest that AP with cefuroxime/metronidazole is less effective in reducing SSO compared with mezlocillin/sulbactam and tazobactam/piperacillin. We assume that the timing of this AP regimen of < 30 min or 30–60 min prior to colorectal surgery does not impact the SSI rate.

## Introduction

Especially in colorectal surgery, postoperative infections and anastomotic leaks are feared. These SSIs are a serious health problem leading to prolonged hospital stay, increased postoperative morbidity, and costs^1,2^. The incidence of SSI after colorectal surgery ranges from 3.6 to 7.1%^3,4^. Naturally, ways are sought to avoid these complications.

It has been revealed that the administration of an antimicrobial prophylaxis (AP) reduces the rate of SSI following colorectal cancer surgery^5–7^. In 1981, Baum et al. conducted the first systematic review on that topic. They compared the rate of SSI with and without AP prior to colon surgery. A reduced rate of SSI was revealed. Hence, Nelson et al. (2014) performed a Cochrane analysis on the topic. The authors reviewed 260 trials (n = 43,451) and 68 different antibiotics^5,6^ and found high quality evidence on AP prior to elective colorectal surgery. A 75%-reduced risk of SSI was estimated by the authors^5^. The Cochrane analysis showed that the optimal timing of AP following colorectal surgery remains unclear.

Recent literature has criticised the definition of SSI as being too narrow to cover the broad phenomenon of wound complications, and extensions have been proposed to categorise wound events not covered by the CDC SSI concept^8^. One of these is the concept of surgical site occurrence (SSO), which includes purulent drainage, stab abscess and seroma^9^. In order to identify clinically relevant SSOs and SSIs, the concept of surgical site infections requiring intervention (SSOPI) was additionally coined.

We conducted this study to make an informed contribution to this discussion. The aim was to find out more precisely when is the right time to administer antibiotics and whether the rate of SSI and SSO after surgery can be reduced.

## Patients and methods

A unicentric retrospective cohort study investigating optimal timing of AP prior to colorectal cancer surgery was conducted.

The files of individuals who underwent colorectal cancer surgery at the University Hospital Brandenburg an der Havel (Germany) between 2009 and 2017 were examined. The data extraction took place from 2018 to 2019. Statistical analysis was conducted in 2020. No digital data could be collected before 2009, as the new hospital computer operating system was introduced in that year. A systematic follow-up did not take place.

The trial was approved by the Ethics Committee of the ‘Ärztekammer Brandenburger’ (Medical Association Brandenburg) in September 2022 (Eth-2022-144-BO-ff) and conducted in accordance with the ethical standards of the Helsinki Declaration 1975. The study was registered with the German clinical trial registry DRKS (DRKS00030355). No funding has been received.

### Objectives

The primary objective was to assess whether different timings of AP (< 30 min, 30–60 min, > 60 min prior to surgery, AP after surgery) had an effect on surgical outcomes following colorectal surgery. Primary endpoint was the rate of occurrence (yes vs no) and grade (superficial vs. deep vs. organ/space) of SSI according to the Centers for Disease Control and Prevention Guideline for the Prevention of Surgical Site Infection following colorectal surgery. The diagnosis was made clinically and radiologically.

Secondary objectives endpoints were the rate of wound dehiscence, burst abdomen, and re-operations due to surgical site occurrences (SSO), more specifically wound dehiscence, burst abdomen, perineal wound healing disorder and seroma formation. Furthermore, the frequency of re-operations due to surgical site occurrences was assessed (SSOPI). The standard wound documentation by the medical staff was used for wound grading (SSO, SSOPI). Seroma formation was diagnosed clinically. No ultrasound imaging took place^8^.

### Inclusion criteria

Electronic files of individuals who were electively operated on due to colorectal cancer with an open surgical approach (midline incision) were analyzed.

### Exclusion criteria

Patients who received neither cefuroxime/metronidazole nor piperacillin/tazobactam nor mezlocillin/sulbactam were excluded. Also Individuals who didn’t receive any AP or received it after incision were excluded.

### Surgical procedures

The operations performed were a left and right hemicolectomy, a sigmoid resection, a deep anterior rectal resection and a rectal extirpation.

### AP-regimen

Over the years, AP regimes in hospitals have been changed according to an adapted protocol of our hospital. The decision was mainly influenced by national guidelines and recommendations of the Paul Ehrlich Institute (Germany) as well as clinical experience and costs^10,11^.

### Statistics

From the database following variables were extracted: age at the time of surgery, sex, BMI (in kg/m^2^), location of carcinoma (colon vs. rectum vs. colorectal), ASA class, administered AP regimen, timing of AP, SSI grading, SSO categories and SSOPI.

Timing of AP was grouped in four categories in relation to timepoint of surgery (more than 60 min, 30–60 min, less than 30 min prior to surgery and after surgery). Three different AP regimens were administered during the study period (Cefuroxime 1.5 g/metronidazole 0.5 g, mezlocillin 2 g/sulbactam 0.5 g, piperacillin 4 g/tazobactam 0.5 g.

Univariable distribution of Quantitative quantitative data variables were examined graphically by histograms and numerically by calculating skewness and excess and their 95%-confidence intervals. If confidence intervals of skewness and excess both includes 0, then a variable is considered to be normally distributed and reported using the mean and standard deviation (SD). Otherwise the median and interquartile range (IQR) are used. Categorical expressed as mean ± standard deviation (SD) or median with interquartile range (IQR), and qualitative data variables were generally reported as frequency and percentage.

Baseline comparison of patient characteristic between the three different AP regimens as well as timing of AP were performed. Categorical variables were compared using Fishers’ Exact test. As continuous variables were not normally distributed within each factor level, Kruskal–Wallis-Tests were used to compare continuous variables between AP regimens and AP timings.

Statistical analyses were conducted using R (version 4.2.1., The R Foundation, Vienna, Austria). All tests were two-sided and *p* values < 0.05 were considered indicative of statistical significance. Chi-squared tests and Kruskal–Wallis-Test were used to determine whether the different groups of AP (mezlocillin/sulbactam, tazobactam/piperacillin, and cefuroxime/metronidazole) differed in categorical (sex, ASA stage) and continuous (age, BMI) baseline characteristics. Primary and secondary endpoint variabless were compared between AP groups and groups of different AP timings.

Multiple binary logistic regression models were performed to identify risk factors for SSIs, SSOs and SSOPIs. One overall regression model that incorporated AP group regimen as a factor and three models stratified by AP group regimen were conducted. Models were adjusted for age, sex, ASA-status, diagnosiscarcinoma location, and A regimen. As no SSI occurred in some factor levels, which may result in non-convergence in a maximum-likelihood estimation, a bias correction according to Firth et al.^12^ took place^10^. Initially it was planned to include BMI as covariate in multivariable analysis but because of the large proportion of missing data (n = 192) it was only considered within a sensitivity analysis. No significance adjustment for multiple models were performed.

Statistical analyses were conducted using R (version 4.2.1., The R Foundation, Vienna, Austria). All tests were two-sided and *p* values < 0.05 were considered indicative of statistical significance.

### Ethical approval

This retrospective analysis was conducted according to the German ethics law and was approved by the Ethics Board of Landesärztekammer Brandenburg (Germany).

### Informed consent

Due to the nature of this retrospective study and the preserved anonymity of patients, a waiver of informed consent was obtained from (University hospital Brandenburg/Havel, Germany).

## Results

A total of 531 patient files of individuals who underwent elective open colorectal cancer surgery were analyzed. The median age of patients at the time of their operation was 73 years (IQR 64–80). A total of 306 individuals were male and 225 were females. The median BMI among these individuals was 26.6 kg/m^2^ (IQR 24.0–29.4; missing: 192). 94 (17.7%) individuals had an ASA-Status I, 253 (47.6%) an ASA-Status II, and 184 (34.7%) an ASA-Status III. In terms of diagnoses, 325 (61.2%) individuals suffered from a colon carcinoma, 204 (38.4%) from a rectum carcinoma, and 2 patients (0.4%) from colon and rectum carcinoma.

A rectum extirpation took place in 18 cases (3.3%). A total of 3 different AP regimens were used (mezlocillin/sulbactam, n = 191 (36%); tazobactam/piperacillin, n = 114 (21.5%); cefuroxime/metronidazole, n = 226 (42.6%). In terms of perioperative AP timing, 326 (61.4%) individuals received the medication < 30 min before surgery, 166 (31.3%) between 30 and 60 min of surgery, 22 (4.1%) more than 1 h before surgery, and 15 (2.6%) after incision. In two cases timing was not stated.

Regarding SSO of midline incision (n = 44), 11 (2.1%) patients suffered from a burst abdomen, 6 (1.1%) from a seroma, 7 (1.3%) from wound dehiscence, and in one case it was not stated. In 19 cases a SSI of the midline incision was diagnosed (Grade I, n = 13 (2.4%); grade II, n = 2 (0.3%), grade III, n = 4 (0.7%). In 17 (3.2%) individuals a SSOPI was stated.

### Univariate analysis AP groups and AP timing

The AP groups mezlocillin/sulbactam, tazobactam/piperacillin, and cefuroxime/metronidazole did not differ in age, gender, BMI, and perioperative data (Table 1). Patients in the cefuroxime/metronidazole group had significantly more often ASA-I status. The frequency of SSIs and SSOs did not differ significantly between AP groups, neither when collapsed across SSI grades (*p* = 0.065), nor comparing different SSI grades (*p* = 0.24). With significance, patients among the cefuroxime/metronidazole group had the highest SSOPI rate. Significant univariate differences in AP timing between AP regimens (*p* = 0.040) were due to “not stated” cases and dropped to non-significance after exclusion of these patients (*p* = 0.051).

**Table 1:** Univariate analysis on different AP groups.

Variable	n = 531^1^	Mezlocillin/Sulbactam n = 191^1^	Cefuroxim/Metronidazole n = 226^1^	Tazobactam/Piperacillin n = 114^1^	*p*-value^2^
Sex					0.60
Male	306 (57.6%)	115 (60.2%)	125 (55.3%)	66 (57.9%)	
Female	225 (42.4%)	76 (39.8%)	101 (44.7%)	48 (42.1%)	
Age (years)	73 (64–80)	72 (65–78)	75 (65–81)	73 (64–80)	0.059
BMI (kg/m^2^)	26.6 (24.0–29.4)	27.7 (25.1–29.8)	26.5 (23.6–29.4)	26.1 (24–29.4)	0.34
Missing:	192	147	15	30	
ASA-Status					** < 0.001**
I	94 (17.7%)	14 (7.3%)	69 (30.5%)	11 (9.6%)	
II	253 (47.6%)	106 (55.5%)	84 (37.2%)	63 (55.3%)	
III	184 (34.7%)	71 (37.2%)	73 (32.3%)	40 (35.1%)	
Carcinoma location					0.092
Colon carcinoma	325 (61.2%)	105 (55%)	146 (64.6%)	74 (64.9%)	
Colon and rectum carcinoma	2 (0.4%)	2 (1%)	0 (0%)	0 (0%)	
Rectum carcinoma	204 (38.4%)	84 (44%)	80 (35.4%)	40 (35.1%)	
Surgical approach					
Rectum extirpation	18 (3.4%)	4 (2.1%)	14 (6.2%)	0 (0%)	**0.004**
Perioperative AP timing					**0.040**
< 30 min	326 (61.4%)	102 (53.4%)	149 (65.8%)	75 (65.8%)	
30–60 min	166 (31.3%)	65 (34%)	68 (30.1%)	33 (28.9%)	
> 60 min	22 (4.1%)	13 (6.8%)	5 (2.2%)	4 (3.5%)	
After incision	15 (2.8%)	9 (4.7%)	4 (1.8%)	2 (1.8%)	
Not stated	2 (0.4%)	2 (1.0%)	0 (0%)	0 (0%)	
SSO of midline incision					0.11
Burst abdomen	11 (2.1%)	1 (0.5%)	7 (3.1%)	3 (2.6%)	
Seroma	6 (1.1%)	1 (0.5%)	5 (2.2%)	0 (0.0%)	
Wound dehiscence	7 (1.3%)	4 (2.1%)	3 (1.3%)	0 (0.0%)	
Perineal wound healing disorder	2 (0.4%)	1 (0.5%)	0 (0.0%)	1 (0.9%)	
SSI					0.24
Grade I	13 (2.4%)	3 (1.6%)	7 (3.1%)	3 (2.6%)	
Grade II	2 (0.4%)	0 (0.0%)	2 (0.9%)	0 (0.0%)	
Grade III	4 (0.8%)	0 (0.0%)	4 (1.8%)	0 (0.0%)	
SSOPI	17 (3.2%)	1 (0.5%)	12 (5.3%)	4 (3.5%)	**0.010**

AP, Antimicrobial prophylaxis; ASA-Status American Society of Anesthesiology; BMI, Body mass index; SSI, Surgical site infection; SSO, Surgical site occurrence; SSOPI, Surgical site occurrences requiring procedural interventions.

^1^n (%); Median (IQR).

^2^*p*-values of Kruskal–Wallis-Test and Fishers’ Exact Test for continuous and categorical variables, respectively. Significant values are in bold.

Differences in study variables regarding different AP timings are summarized in Supplementary Table S1. No baseline differences in patient characteristics were observed. The univariate analysis on the effect of AP timing showed no significant differences SSI (*p* = 0.34), SSO (*p* = 0.97) and SSOPI (*p* = 0.71) was observed.

### Multivariate analysis on SSI

The result of the logistic regression model for the primary endpoint of SSI is summarized in Table 2. Different timing of AP was not identified as an independent risk factor for SSI occurrence. However, it can be observed that the odds ratios tend to increase with prolonged time between AP and surgery.

**Table 2:** Multivariate analysis on SSI.

Variable	N	Event N	OR^1^	95% CI^1^	*p*-value
AP regimen					
Cefuroxim/metronidazole	226	13	–	–	
Mezlocillin/sulbactam	191	3	0.22	0.07–0.70	**0.010**
Tazobactam/piperacillin	114	3	0.43	0.14–1.36	0.10
Sex					
Male	306	7	–	–	
Female	225	12	2.42	1.02–5.75	**0.045**
Age	531	19	0.97	0.93–1.01	0.19
ASA-status					
I	94	1	–	–	
II	253	7	3.79	0.72–20.1	0.12
III	184	11	8.6	1.59–46.5	**0.012**
Diagnosis					
Colon carcinoma	325	10	–	–	
Rectum carcinoma	204	9	1.67	0.69–4.01	0.25
Colon and rectum carcinoma	2	0	12.4	0.26–596	0.20
Perioperative AP timing					
< 30 min	326	10	–	–	
30–60 min	166	8	1.42	0.58–3.46	0.45
> 60 min	22	1	2.33	0.35–15.4	0.38
After incision	15	0	0.85	0.04–16.0	0.91
Not stated	2	0	20.7	0.47–1.05	0.13
Null deviance			164		
Null distribution			530		
Log-likelihood			− 73.1		
Akaike Information Criterion			172		
Bayesian information criterion			228		
Deviance			146		
Residual df			518		
No. observation			531		

^1^OR, Odds Ratio.

AP, Antimicrobial prophylaxis; ASA-Status American Society of Anesthesiology; BMI, Body mass index; CI, Confidence interval; SSI, Surgical site infection. Significant values are in bold.

The likelihood of SSI was lower when patients received mezlocillin/sulbactam in comparison to cefuroxime/metronidazole (OR = 0.22, 95% CI 0.07–0.70, *p* = 0.010). Females had a higher probability of suffering from an SSI than males (OR 2.42, CI 1.02–5.75, *p* = 0.045). With significance, the ASA-Status III (OR 8.6, CI 1.59–46.5, *p* = 0.012) had a higher risk for SSI occurrence than ASA-Status I and II. Within the sensitivity analyses, where BMI was additionally considered as covariate, results regarding the effect of AP timing on SSI occurrence were not altered. Separate logistic regression models, stratified for the three AP regimens, generally suffered from rarity of SSI events, resulting in high standard errors and wide confidence intervals (Supplementary Table S2). Only in the model for the cefuroxime/metronidazole AP regimen enough events occurred to obtain stable estimates. Here, the same trend as in the overall analysis was observed, with highest Odds ratios in the group with AP more than 60 min before incision.

Regarding secondary endpoints, SSO and SSOPI, multivariable logistic regression models (Supplementary Table S3) showed no effect of AP timing (*p*’s > 0.99). For SSO, ASA-Score (*p* = 0.027) and AP regimen (*p* = 0.016) were the only significant predictors, with highest SSO-rate for ASA-Status III and the cefuroxime/metronidazole AP. For SSOPI only the AP regimen was identified as significant predictor (*p* = 0.023), with lowest SSOPI-rate in the mezlocillin/sulbactam group.

## Discussion

In principle, the antibiotics administered covered the expected spectrum of germs. The antibiotic regimens used were Piperacillin/Tazobactam, Cefuroxime/Metronidazole and Mezlocillin/Sulbactam.

Piperacillin/tazobactan and mezlocillin/sulbactam are antibiotics with short half-lives of 45 and 60 min, respectively. Cefuroxime has a half-life of 60 min and Metrodnidazole of 7 h. The latter combination therefore has the advantage that an antibiotic with a short half-life has been combined with an antibiotic with a longer half-life, so that in our opinion there is sufficiently better protection so far^10,11^.

Based on the results, we are beginning to reconsider this, especially if further studies confirm this and we have two more potent antibiotic combinations in reserve based on the results of our study. In all regimens, a repeat dose was given if the surgery time was longer than 2 h. An additional risk factor was a blood loss of more than 1000 ml. These additional antibiotic doses were not separately recorded.

The optimal timing of AP after colorectal surgery is still a matter of debate. A Cochrane analysis in 2014 did not lead to a clear recommendation in this regard^5^. Hence we conducted the retrospective analysis at hand to reveal more evidence on that topic. Different timing of AP (< 30 min vs. 30–60 min vs. more than 1 h prior to surgery vs. after incision) was not identified as an independent risk factor of SSIs in a multivariable logistic regression.

As already mentioned above, the following operations were performed: left and right hemicolectomy, sigmoid resection, deep anterior rectal resection and rectal extirpation. In this context, it should of course be mentioned that the different operations performed also have an influence on secondary infections. The literature search confirms that a greater number of wound infections were found after rectal resections. Goto et al. (2016) show this impressively for laparoscopic rectal surgery compared to laparoscopic colon surgery^13^. This was also confirmed by Konishi et al. (2006) for all surgical techniques. Here, too, rectal procedures showed a significantly higher rate of surgical concomitant infections^14,15^.

**Figure 1:**
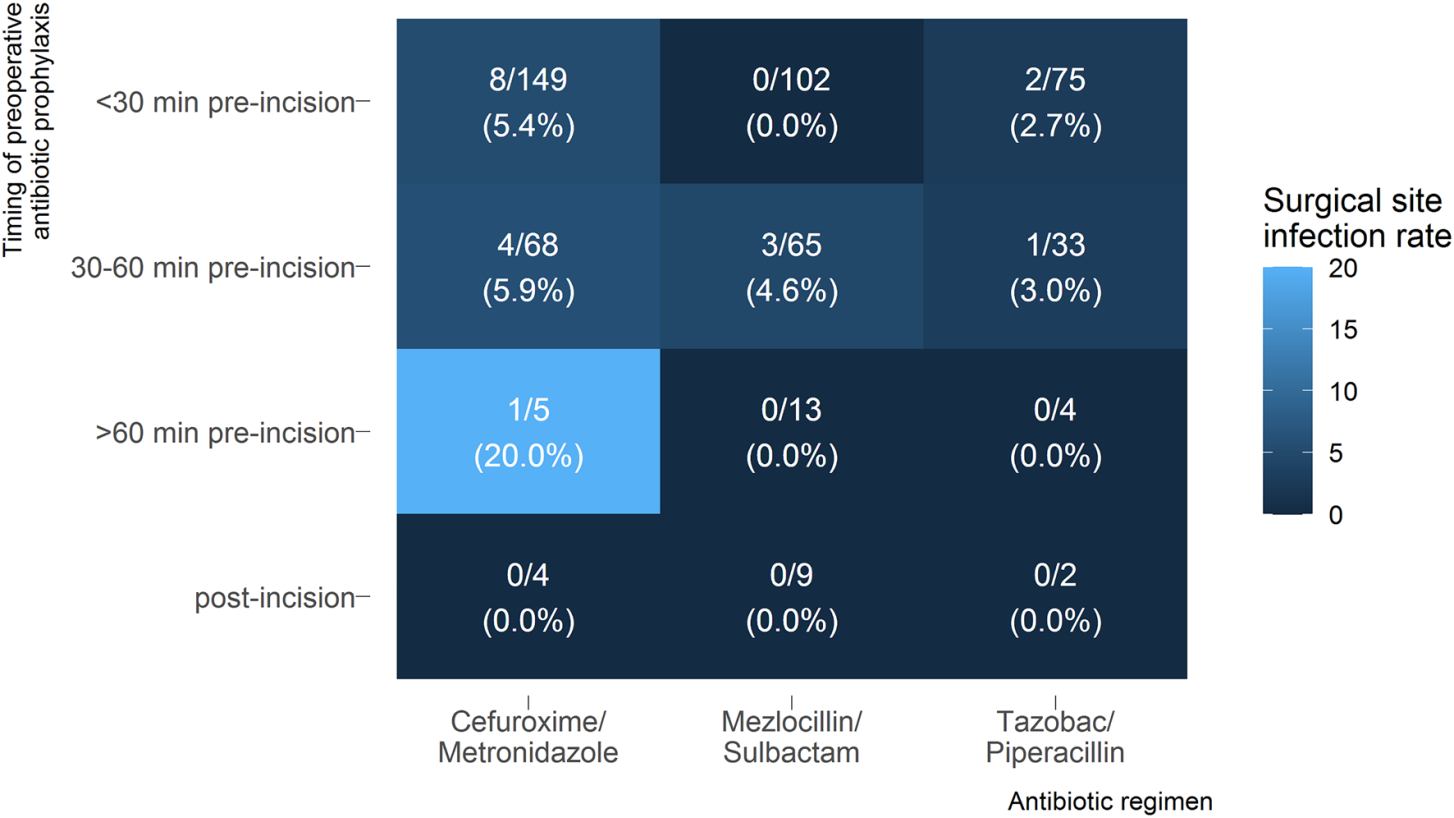
Timing of preoperative antibiotic prophylaxis.

A total of 22 patients received AP more than hour prior to surgery. One SSI occurred in these individuals (Fig. 1). Generally, a trend to elevated SSI rates with increasing delay between AP and surgery was observed descriptively. Without reaching significance the likelihood of this event was increased with an OR of 2.33, when this (early) timing was chosen in comparison to AP < 30 min (OR 2.33, CI 0.35–15.4,* p* = 0.38; Table 2). Lack of significance of the AP timing factor may be explained by the small sample size. Hence, there might be an increased risk of SSIs, when the AP is given too early. In general, different half-lives of antibiotics should be taken into account. As an example, the half-life of cefuroxime is only 1 hour^16^. It is conceivable and logical that the antimicrobial effect decreases if administered too early.

In summary, our results suggest that the AP should be administered within 1 h before colorectal surgery. That is consistent with published recommendations on AP timing following other surgical procedures (cardiac, hip/knee arthroplasty, and hysterectomy cases)^17^.

Cefuroxime is currently the most commonly used antibiotic in surgical prophylaxis and is often given in combination with metronidazole in order to cover the anaerobic bacteria spectrum^5^. When conducting a univariate analysis on the different AP groups, we revealed no different rates of SSIs in any particular group; but the SSI grade was higher among individuals who received cefuroxime/metronidazole. This higher grade SSI, such as deep wound infections, may also lead to wound dehiscence and burst abdomen, or so called SSOs. In that respect, the SSO and SSOPI rates occurred significantly more frequently in individuals receiving cefuroxime and metronidazole (Table 1). This may indicate a minor efficiency on reducing SSI, SSO, and SSOPI rates of this AP in comparison to mezlocillin/sulbactam or tazobactam/piperacillin administering.

The following further observations may confirm this assumption. Patients in the mezlocillin/sulbactam and tazobactam/piperacillin AP group had a higher morbidity (ASA status I: n = 14 (7.3%) and n = 11 (9.6%) vs. n = 69 (30.5%), *p* < 0.001; Table 1). As presumed, the antimicrobial effect decreases if AP is administered too early. In our patients, mezlocillin/sulbactam and tazobactam/piperacillin were more frequently administered more than 1 h before surgery than in patients receiving cefuroxime/metronidazole (n = 13 (6.8%) and n = 4 (3.5%) vs. 5 (2.2%), Table 1). Although patients in the cefuroxime/metronidazole group suffered from less morbidity and are most likely to benefit from more optimal AP timing, SSOs with higher grade SSIs and SSOPIs appeared more often.

Furthermore, of the 22 individuals who received AP more than an hour prior to surgery, one SSI occurred. Cefuroxime/metronidazole was administered to this patient. Only 4 other individuals received this AP regimen more than an hour prior to surgery. This means that 20% of this group suffered from a SSI (Fig. 1), an estimate with very high uncertainty.

Some trials were conducted on that topic. Ambrose et al. (1983) did not detect significant differences in terms of SSIs when comparing mezlocillin with cefuroxime/metronidazole in a prospective randomized clinical trial^18^. Stubbs et al. (1987) and Diamond et al. (1988) could also not reveal any differences regarding SSI rates between mezlocillin/metronidazole and cefuroxime/metronidazole^19,20^. The aforementioned publications were on randomized clinical trials with a higher statistical power than those of our retrospective analysis. But the study groups were not exactly comparable to our patients in terms of AP timing, doses, and AP regimens. Unfortunately, our review of the literature did not reveal any publications comparing the same AP regimens (mezlocillin/sulbactam vs. tazobactam/piperacillin vs. cefuroxime/metronidazole), nor did the Cochrane analysis by Nelson in 2014^5,21^.

In summary, a minor efficiency of AP with cefuroxime/metronidazole on reducing SSI, SSO, and SSOPI rates compared to mezlocillin/sulbactam and tazobactam/piperacillin can be assumed. Further trials are mandatory.

The multivariate analysis revealed that women had a higher risk of SSI occurrence (n = 225, 12 events, OR 2.42, CI 1.02–5.75, *p* = 0.045; Table 2). These findings seem to be in contrast to current knowledge. To that, Xu et al. (2021) performed a meta-analysis on risk factors for surgical site infections in individuals undergoing colorectal surgery. A total of 31 trials were analyzed. With moderate quality a correlation of SSI with male sex was reported^22^. A multiple-center prospective study of 3,663 consecutive patients in China conducted by Hou et al. (2020) did not reveal female sex as a risk factor for SSIs^3^. Since the mentioned publications are from China, the various risk profile in terms of gender might be caused by social, economic, and racial differences. Further research is needed.

In addition to antibiotic prophylaxis, other measures have also been discussed in recent years. The focus is on preoperative bowel cleansing, own hair removal, glycaemic control and normothermia. These measures have been shown to significantly reduce SSI and should now be incorporated into the surgical approach^22–25^. However, this has not yet been implemented. In the context of the results of this study, we plan to apply it in the future.

Our analysis has limitations. The retrospective study design without systematic follow-up must be taken into account.Furthermore, some SSIs might not be diagnosed, as patients were treated elsewhere after discharge from our hospital. A total of 192 patient’s files lacked information on BMI, thus multivariate analysis was conducted without this parameter. The rate of seroma formation was low but the diagnosis was made clinically. No ultrasound imaging took place. Hence, a higher rate might be expected. Although wound documentation by medical staff is mandatory in Germany, some SSIs might be overseen or misinterpreted. Another limitation of the study is the fact that we have no data for the period before 2009. Unfortunately, the hospital's computer system was renewed this year. The data before that are no longer available. We determined an SSI rate of 3.6%, which is consistent with published rates^3,17^.

## Conclusion

Our results suggest that AP with cefuroxime/metronidazole is less effective in reducing SSO and SSOPI rates compared with mezlocillin/sulbactam and tazobactam/piperacillin. We further assume that the timing of this AP regimen of < 30 min or 30–60 min prior to colorectal surgery does not impact the SSI rate, although larger prospectively randomized studies with sufficient statistical power are required to assess the effect systematically.

## Supplementary Information

Supplementary Table 1.

Supplementary Table 2.

Supplementary Table 3.

## Data Availability

The datasets used and/or analyzed during the current study available from the corresponding author on reasonable request.
