# Author Correction: Optimal timing for antimicrobial prophylaxis to reduce surgical site infections: a retrospective analysis of 531 patients

**DOI:** 10.1038/s41598-023-37415-3

**Published:** 2023-06-27

**Authors:** Christoph Paasch, Claus Schildberg, Sebastian Lünse, Sophie Heisler, Jens Meyer, Jette Kirbach, Elisa Kobelt, Richard Hunger, Isabel-Elena Haller, Chrissanthi Helmke, Rene Mantke

**Affiliations:** 1 Department of Surgery, Brandenburg Medical School, University Hospital Brandenburg/Havel, 14770 Brandenburg, Germany; 2 Clinic for General and Visceral Surgery, Klinikum Magdeburg gGmbH, Magdeburg, Germany; 3 Faculty of Health Science Brandenburg, Brandenburg Medical School, University Hospital Brandenburg/Havel, 14770 Brandenburg, Germany; 4 Clinic for General and Visceral Surgery, University Hospital Brandenburg an der Havel, Brandenburg Medical University, Hochstraße 29, 14770 Brandenburg an der Havel, Germany

Correction to: *Scientific Reports* 10.1038/s41598-023-36588-1, published online 09 June 2023

In the original version of this Article Christoph Paasch and Claus Schildberg were omitted as equally contributing authors.

The original Article has been corrected.

