# Supplementary material for: Optimal timing for antimicrobial prophylaxis to reduce surgical site infections: a retrospective analysis of 531 patients
Source: Sci Rep. 2023 Jun 9;13:9405. doi: 10.1038/s41598-023-36588-1 (PMC10256713; doi:10.1038/s41598-023-36588-1)
Supplement: Supplementary file 1 — Supplementary Table 1. [file 41598_2023_36588_MOESM1_ESM.docx]

Table S1: Summary of study variables according to AP timing

| **Variable** | **Overall**  N = 529^1^ | **<30 min pre-incision**  N = 326^1^ | **30-60 min pre-incision**  N = 166^1^ | **>60 min pre-incision**  N = 22^1^ | **post-incision**  N = 15^1^ | ***p*-value**^2^ |
| --- | --- | --- | --- | --- | --- | --- |
| AP group |  |  |  |  |  | 0.051 |
| Cefuroxime/Metronidazole | 226 (42.7%) | 149 (45.7%) | 68 (41%) | 5 (22.7%) | 4 (26.7%) |  |
| Mezlocillin/Sulbactam | 189 (35.7%) | 102 (31.3%) | 65 (39.2%) | 13 (59.1%) | 9 (60%) |  |
| Tazobac/Piperacillin | 114 (21.6%) | 75 (23%) | 33 (19.9%) | 4 (18.2%) | 2 (13.3%) |  |
| Sex |  |  |  |  |  | 0.51 |
| male | 305 (57.7%) | 195 (59.8%) | 92 (55.4%) | 11 (50%) | 7 (46.7%) |  |
| female | 224 (42.3%) | 131 (40.2%) | 74 (44.6%) | 11 (50%) | 8 (53.3%) |  |
| Age (years) | 73 (64 – 80) | 74 (64 – 80) | 73 (65 – 80) | 72.5 (65 – 78) | 72 (60 – 80) | 0.78 |
| BMI (kg/m2) | 26.6 (24.0 – 29.4) | 26.1 (23.6 – 2.09) | 27.1 (24.2 – 30.3) | 27.2 (25.2 – 28.3) | 28.8 (25.3 – 29) | 0.19 |
| Missing | 190 | 104 | 63 | 14 | 9 |  |
| ASA-Status |  |  |  |  |  | 0.071 |
| I | 93 (17.6%) | 66 (20.2%) | 22 (13.3%) | 5 (22.7%) | 0 (0.0%) |  |
| II | 252 (47.6%) | 152 (46.6%) | 77 (46.4%) | 12 (54.5%) | 11 (73.3%) |  |
| III | 184 (34.8%) | 108 (33.1%) | 67 (40.4%) | 5 (22.7%) | 4 (26.7%) |  |
| Carcinoma location |  |  |  |  |  | 0.055 |
| Colon carcinoma | 325 (61.4%) | 213 (65.3%) | 94 (56.6%) | 8 (36.4%) | 10 (66.7%) |  |
| Colon and rectum carcinoma | 2 (0.4%) | 1 (0.3%) | 1 (0.6%) | 0 (0.0%) | 0 (0.0%) |  |
| Rectum carcinoma | 202 (38.2%) | 112 (34.4%) | 71 (42.8%) | 14 (63.6%) | 5 (33.3%) |  |
| SSO | 45 (8.5%) | 29 (8.9%) | 14 (8.4%) | 1 (4.5%) | 1 (6.7%) | 0.97 |
| SSO type |  |  |  |  |  | **0.043** |
| Perineal wound healing disorder | 2 (0.4%) | 1 (0.3%) | 0 (0.0%) | 0 (0.0%) | 1 (6.7%) |  |
| Burst abdomen | 11 (2.1%) | 10 (3.1%) | 1 (0.6%) | 0 (0.0%) | 0 (0.0%) |  |
| Seroma | 6 (1.1%) | 4 (1.2%) | 2 (1.2%) | 0 (0.0%) | 0 (0.0%) |  |
| Wound dehiscence | 7 (1.3%) | 4 (1.2%) | 3 (1.8%) | 0 (0.0%) | 0 (0.0%) |  |
| SSI |  |  |  |  |  | 0.34 |
| Grade I | 13 (2.5%) | 8 (2.5%) | 5 (3.0%) | 0 (0.0%) | 0 (0.0%) |  |
| Grade II | 2 (0.4%) | 1 (0.3%) | 1 (0.6%) | 0 (0.0%) | 0 (0.0%) |  |
| Grade III | 4 (0.8%) | 1 (0.3%) | 2 (1.2%) | 1 (4.5%) | 0 (0.0%) |  |
| SSOPI | 17 (3.2%) | 12 (3.7%) | 4 (2.4%) | 1 (4.5%) | 0 (0.0%) | 0.71 |
| ^1^ n (%); Median (IQR) | | | | | | |
| ^2^ P-values of Kruskal-Wallis-Test and Fishers’ Exact Test for continuous and categorical variables, respectively | | | | | | |
