# Supplementary material for: Optimal timing for antimicrobial prophylaxis to reduce surgical site infections: a retrospective analysis of 531 patients
Source: Sci Rep. 2023 Jun 9;13:9405. doi: 10.1038/s41598-023-36588-1 (PMC10256713; doi:10.1038/s41598-023-36588-1)
Supplement: Supplementary file 2 — Supplementary Table 2. [file 41598_2023_36588_MOESM2_ESM.docx]

Table S2: Logistic regression models for primary endpoint (Surgical site infection), stratified by antibiotic prophylaxis regimen

|  | **Cefuroxime/ Metronidazole** | | | | | **Mezlocillin/ Sulbactam** | | | | | **Tazobac/ Piperacillin** | | | | |
| --- | --- | --- | --- | --- | --- | --- | --- | --- | --- | --- | --- | --- | --- | --- | --- |
| **Variable** | **N** | **Event N** | **OR**^1^ | **95% CI**^1^ | **p-value** | **N** | **Event N** | **OR**^1^ | **95% CI**^1^ | **p-value** | **N** | **Event N** | **OR**^1^ | **95% CI**^1^ | **p-value** |
| Sex | 226 | 13 |  |  |  | 191 | 3 |  |  |  | 114 | 3 |  |  |  |
| Male | 125 | 5 | — | — |  | 115 | 1 | — | — |  | 66 | 1 | — | — |  |
| Female | 101 | 8 | 2,02 | 0,70 – 5,82 | 0,19 | 76 | 2 | 2,58 | 0,48 – 13,8 | 0,27 | 48 | 2 | 2,85 | 0,50 – 16,3 | 0,24 |
| Age | 226 | 13 | 0,98 | 0,93 – 1,04 | 0,52 | 191 | 3 | 0,96 | 0,88 – 1,04 | 0,27 | 114 | 3 | 1,00 | 0,92 – 1,10 | 0,95 |
| ASA score | 226 | 13 |  |  |  | 191 | 3 |  |  |  | 114 | 3 |  |  |  |
| I | 69 | 1 | — | — |  | 14 | 0 | — | — |  | 11 | 0 | — | — |  |
| II | 84 | 3 | 2,18 | 0,36 – 13,3 | 0,40 | 106 | 1 | 1,24 | 0,04 – 34,6 | 0,90 | 63 | 3 | 2,07 | 0,12 – 35,3 | 0,61 |
| III | 73 | 9 | 7,43 | 1,35 – 41,0 | 0,021 | 71 | 2 | 2,62 | 0,09 – 80,2 | 0,58 | 40 | 0 | 0,48 | 0,01 – 18,0 | 0,69 |
| Carcinoma location | 226 | 13 |  |  |  | 191 | 3 |  |  |  | 114 | 3 |  |  |  |
| Colon carcinoma | 146 | 9 | — | — |  | 105 | 0 | — | — |  | 74 | 1 | — | — |  |
| Rectum carcinoma | 80 | 4 | 0,81 | 0,25 – 2,65 | 0,73 | 84 | 3 | 6,53 | 0,72 – 58,9 | 0,094 | 40 | 2 | 2,68 | 0,49 – 14,7 | 0,26 |
| Colon and rectum carcinoma |  |  |  |  |  | 2 | 0 | 24,3 | 0,28 – 2.105 | 0,16 |  |  |  |  |  |
| Perioperative AP timing | 226 | 13 |  |  |  | 191 | 3 |  |  |  | 114 | 3 |  |  |  |
| <30 minutes | 149 | 8 | — | — |  | 102 | 0 | — | — |  | 75 | 2 | — | — |  |
| 30-60 mininutes | 68 | 4 | 0,99 | 0,31 – 3,17 | >0,99 | 65 | 3 | 7,64 | 0,79 – 74,4 | 0,080 | 33 | 1 | 1,85 | 0,29 – 11,6 | 0,51 |
| >60 minutes | 5 | 1 | 7,19 | 0,64 – 80,5 | 0,11 | 13 | 0 | 3,99 | 0,13 – 123 | 0,43 | 4 | 0 | 1,63 | 0,05 – 50,6 | 0,78 |
| After incision | 4 | 0 | 1,02 | 0,04 – 27,9 | >0,99 | 9 | 0 | 5,80 | 0,11 – 301 | 0,38 | 2 | 0 | 4,85 | 0,08 – 283 | 0,45 |
| Null deviance |  |  | 99,5 |  |  |  |  | 30,9 |  |  |  |  | 27,7 |  |  |
| Null df |  |  | 225 |  |  |  |  | 190 |  |  |  |  | 113 |  |  |
| Log-likelihood |  |  | -44,2 |  |  |  |  | -11,8 |  |  |  |  | -13,1 |  |  |
| AIC |  |  | 106 |  |  |  |  | 45,6 |  |  |  |  | 44,3 |  |  |
| BIC |  |  | 137 |  |  |  |  | 81,4 |  |  |  |  | 68,9 |  |  |
| Deviance |  |  | 88,4 |  |  |  |  | 23,6 |  |  |  |  | 26,3 |  |  |
| Residual df |  |  | 217 |  |  |  |  | 180 |  |  |  |  | 105 |  |  |
| No. Obs. |  |  | 226 |  |  |  |  | 191 |  |  |  |  | 114 |  |  |
| ^1^OR = Odds Ratio, CI = Confidence interval | | | | | | | | | | | | | | | |
