# Supplementary material for: Optimal timing for antimicrobial prophylaxis to reduce surgical site infections: a retrospective analysis of 531 patients
Source: Sci Rep. 2023 Jun 9;13:9405. doi: 10.1038/s41598-023-36588-1 (PMC10256713; doi:10.1038/s41598-023-36588-1)
Supplement: Supplementary file 3 — Supplementary Table 3. [file 41598_2023_36588_MOESM3_ESM.docx]

Table S3: Logistic regression models for secondary endpoints

|  | **Endpoint: SSO** | | | | | **Endpoint SSOPI** | | | | |
| --- | --- | --- | --- | --- | --- | --- | --- | --- | --- | --- |
| **Variable** | **N** | **Event N** | **OR**^1^ | **95% CI**^1^ | **p-value** | **N** | **Event N** | **OR**^1^ | **95% CI**^1^ | **p-value** |
| Sex | 530 | 45 |  |  | >0,99 | 530 | 17 |  |  | >0,99 |
| Male | 305 | 23 | — | — |  | 305 | 10 | — | — |  |
| Female | 225 | 22 | 1,31 | 0,71 – 2,41 |  | 225 | 7 | 0,97 | 0,40 – 2,35 |  |
| Age (years) | 530 | 45 | 0,99 | 0,96 – 1,02 | >0,99 | 530 | 17 | 0,97 | 0,93 – 1,02 | >0,99 |
| ASA Status | 530 | 45 |  |  | **0,027** | 530 | 17 |  |  | 0,19 |
| I | 94 | 6 | — | — |  | 94 | 2 | — | — |  |
| II | 253 | 15 | 1,52 | 0,57 – 4,03 |  | 253 | 5 | 1,65 | 0,39 – 6,95 |  |
| III | 183 | 24 | 3,52 | 1,32 – 9,38 |  | 183 | 10 | 4,97 | 1,18 – 20,9 |  |
| Carcinoma location | 530 | 45 |  |  | 0,27 | 530 | 17 |  |  | >0,99 |
| Colon and rectum carcinoma | 2 | 0 | — | — |  | 2 | 0 | — | — |  |
| Colon carcinoma | 324 | 22 | 0,23 | 0,01 – 10,4 |  | 324 | 10 | 0,03 | 0,00 – 1,83 |  |
| Rectum carcinoma | 204 | 23 | 0,46 | 0,01 – 20,7 |  | 204 | 7 | 0,04 | 0,00 – 2,55 |  |
| AP regimen | 530 | 45 |  |  | **0,016** | 530 | 17 |  |  | **0,023** |
| Cefuroxime/Metronidazole | 226 | 28 | — | — |  | 226 | 12 | — | — |  |
| Mezlocillin/Sulbactam | 191 | 9 | 0,34 | 0,16 – 0,73 |  | 191 | 1 | 0,11 | 0,02 – 0,52 |  |
| Tazobac/Piperacillin | 113 | 8 | 0,38 | 0,16 – 0,92 |  | 113 | 4 | 0,62 | 0,21 – 1,77 |  |
| Perioperative AP timing | 530 | 45 |  |  | >0,99 | 530 | 17 |  |  | >0,99 |
| <30 minutes | 326 | 29 | — | — |  | 326 | 12 | — | — |  |
| 30-60 minutes | 165 | 14 | 0,87 | 0,45 – 1,70 |  | 165 | 4 | 0,64 | 0,23 – 1,82 |  |
| >60 minutes | 22 | 1 | 0,76 | 0,13 – 4,46 |  | 22 | 1 | 2,99 | 0,48 – 18,6 |  |
| After incision | 15 | 1 | 1,13 | 0,18 – 7,10 |  | 15 | 0 | 0,89 | 0,04 – 18,0 |  |
| Null deviance |  |  | 303 |  |  |  |  | 150 |  |  |
| Null df |  |  | 529 |  |  |  |  | 529 |  |  |
| Log-likelihood |  |  | -141 |  |  |  |  | -67,9 |  |  |
| AIC |  |  | 309 |  |  |  |  | 162 |  |  |
| BIC |  |  | 364 |  |  |  |  | 217 |  |  |
| Deviance |  |  | 283 |  |  |  |  | 136 |  |  |
| Residual df |  |  | 517 |  |  |  |  | 517 |  |  |
| No. Obs. |  |  | 530 |  |  |  |  | 530 |  |  |
| ^1^OR = Odds Ratio, CI = Confidence interval, SSO = Surgical site occurrence, SSOPI = Surgical site occurrence requiring procedural intervention | | | | | | | | | | |
